# Influence of PESU/PVP based nanofibrous separators on the properties of LNMO cathode based Li-ion batteries

**DOI:** 10.1039/d5ra09254j

**Published:** 2026-03-17

**Authors:** T. Kazda, M. Sedlaříková, D. Pleha, J. Tichý, P. Vyroubal, J. Máca, P. Čudek, D. Capková, A. Visintin

**Affiliations:** a Department of Electrical and Electronic Technology, Faculty of Electrical Engineering and Communication, BUT Technická 10 616 00 Brno Czech Republic kazda@vutbr.cz +420 54114 6177; b Instituto de Investigaciones Fisicoquímicas Teóricas y Aplicadas (INIFTA), UNLP, CCT La Plata-CONICET CC 16, Suc. 4 La Plata CP 1900, Argentina; c Department of Chemical Sciences and Bernal Institute, University of Limerick Limerick V94 T9PX Ireland

## Abstract

The properties of selected separators and their influence on the electrochemical properties of a high-voltage cathode material are investigated in this paper. The LiCr_0.1_Ni_0.4_Mn_1.5_O_4_ material synthesized by a solid-state reaction was chosen as the testing material. Four kinds of separators were selected for this study, including a glass fiber separator, a commercial separator made by Celgard and experimental separators made by the Nafigate Corporation. The main goal was to study the properties of the commercial and non-commercial separators and their influence on reached capacity, stability during cycling at high loads and stability during cycling at high temperatures. It was observed that the Nafigate separators are more thermally stable with lower shrinking up to 150 °C and exhibit comparable conductivity to the Celgard separator. In combination with the LiCr_0.1_Ni_0.4_Mn_1.5_O_4_ cathode, the Nafigate separator also achieves higher capacity at lower C-rate (0.5C ∼130 mAh g^−1^) and at higher load (5C) it reaches ∼111 mAh g^−1^. The Celgard separator reaches a capacity of ∼118 mAh g^−1^ at 0.5C and ∼83 mAh g^−1^ at higher load (5C).

## Abstract 2

The PESU/PVP electrospun separator enables higher stability of the cell with an LNMO cathode operating over 5 V.

## Graphical abstract

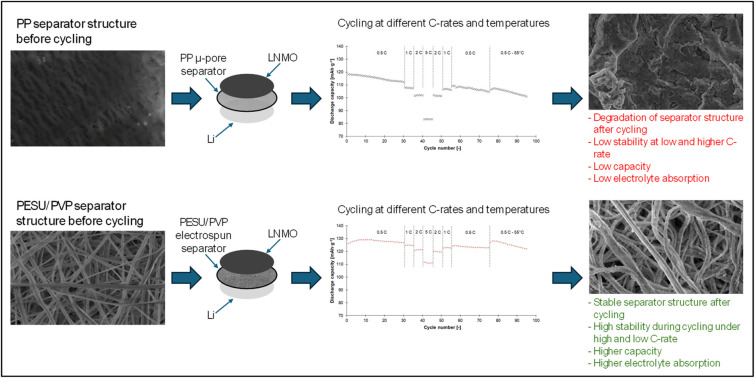

## Introduction

Lithium-ion (Li-ion) batteries are currently the fastest developing type of batteries on the market. In recent years, their production has been growing considerably. For example, 650 tons of cathode materials were produced in 1995, four years after the launch of the first Li-ion batteries on the market by Sony. However, the production of cathode materials increased to 105 000 tons in 2014.^1,2^ The progressing development of wearable electronics, mobile phones and electric cars in recent years was possible thanks to the development of Li-ion batteries. Cathode materials used today will not be able to satisfy the steadily increasing demand for greater amounts of energy stored in batteries. A technology that could increase the capacity of Li-ion batteries in the coming years is the use of high-voltage cathode materials with a discharge plateau potential of about 5 V.^3,4^ One of these materials is also LiNi_0.5_Mn_1.5_O_4_ spinel, which, due to its very stable discharge plateau at 4.7 V *vs.* Li^+^/Li and a theoretical capacity of 148 mAh g^−1^, has a very high gravimetric energy density approaching 700 Wh kg^−1^. This is about 20–30% more than the gravimetric energy density of the currently used cathode materials such as LiFePO_4_ (578 Wh kg^−1^), LiCoO_2_ (510 Wh kg^−1^) and LiMn_2_O_4_ (420 Wh kg^−1^).^5–7^ Another advantage of the higher voltage is that many cells in series can be used to achieve the demanded voltage. However, the disadvantage of this material is the degradation of electrochemical characteristics due to the presence of Mn^3+^ ions, which are closely connected to the lack of oxygen during the preparation at higher temperatures. Ideally, there should be no Mn^3+^ ions present in the compounds, and all the Mn^3+^ ions should be replaced by Ni^2+^ ions. However, a small amount of Mn^3+^ is normally left in the compound after the high temperature treatment. Mn^3+^ ions are easily transformed to Mn^4+^ ions and Mn^2+^ ions (due to the Jahn–Teller effect) that are easily soluble in the electrolyte, particularly at higher temperatures, which leads to loss of capacity during cycling and during storage.^8,9^ One way to avoid this process is the partial replacement of Mn or Ni by another element, such as Cr. Cr^3+^ ions have a higher affinity to oxygen than Mn and Ni, which improves structural stability during cycling.^10,11^ Another advantage of using chromium is that it is electrochemically active on its own thanks to which results in a shift of the discharge plateau to 4.8 V *vs.* Li^+^/Li.^12,13^ Non-electrochemical battery parts used as separators or package may also have a great influence on the final properties of the high voltage battery (reported by Dahn *et al.*^14^ and Chen *et al.*^15^). In this article, the focus is mainly on the main purpose of the separator, which is to serve as a separation layer between the anode and the cathode, preventing a short circuit of the cell. Furthermore, it serves as an electrolyte reservoir by keeping the necessary amount of electrolyte, allowing the battery to function. The separator, due to its material and structural properties, then affects the battery performance, including its durability, safety, energy density and power density.^16,17^ The correct choice of separator for the battery system can affect a wide range of properties, including its cost. The price of the separator currently constitutes ∼20% of the battery cost^18^

The most important requirements for separators are their chemical and electrochemical stability, thickness, good wettability, thermal stability, dimensional stability and sufficient porosity.^16,17,19^ The thickness of the separators is usually between 20 and 25 µm. A thinner separator is better for applications with higher energy density (7–15 µm) and higher power (20–30 µm); however, the chance of its penetration increases at the same time, and thus the risk of battery damage increases.^19,20^ A separator with a higher thickness of 40 µm is required for some applications for safety reasons.^16,19^ Another important property of the separator is porosity. Porosity should be in the range of 40–70% and it should be uniform. Such non-uniformity could lead to non-uniformity of the current distribution and thereby degrade the battery properties.^16,19–21^ The separator must be stable in contact with solvents and materials of the electrodes. Other important structural properties of the separators are shrinkage and consequently, thermal stability. The separator should maintain its dimensional stability at the high thermal load that occurs when the battery is warming up during production, as it must be sufficiently dried. The separator has to withstand a thermal load of 90 °C for 1 h without more than 5% reduction in the dimensions measured in all axes.^16,19^ This high temperature load is further related to the temperature stability of the separator. The separator should separate the anode and cathode materials for as long as possible, thereby preventing the battery from short-circuiting and the reaction of these materials with each other. For this safety reason, a higher melting point of the separator is very important. It should be higher than 150 °C.^16,19^ Another important parameter of separators is the MacMullin number, which expresses a number indicating the increase in resistance of the electrolyte between electrodes, caused by the presence of a separator. Typically, the MacMullin number for commercial separators for Li-ion batteries is between 5 and 15.^20^ The wettability of the separator can be described by contact angle and electrolyte uptake. The contact angle affects the rate and extent of wetting of the separator pores by the electrolyte, which is critical for consistent ion transport. The lower the contact angle, the better the wettability of the separator (∼25° for glass fiber and between 45 to 80° for polypropylene). Electrolyte uptake affects the amount of electrolyte retained in the separator, which affects internal resistance, charging rate, and lifespan. The higher the electrolyte uptake, the higher the electrochemical capacity (400–900% for glass fiber and ∼100% for polypropylene).^22^ The mechanical performance of separators is evaluated based on tensile strength in both the machine and transverse directions, tear resistance, and puncture strength. It is essential for separators to possess sufficient mechanical strength, particularly along the machine direction, to endure the stresses encountered during battery assembly and throughout cycling. Tensile strength measures the force needed to stretch the separator until it breaks, with a typical minimum requirement of around 100 MPa for commercial applications. Tear resistance indicates the ability of the separator to resist tearing or cracking under stress. Puncture strength reflects the separator's capacity to withstand penetration from electrode particulates or sharp lithium dendrites; it is defined as the maximum force a needle must exert to pierce the separator, usually determined using a tensile tester. For commercial use, a puncture strength of at least 300 g is generally required.^22,23^

In terms of structure, the separators can be divided into several categories: microporous membrane separators, non-woven separators, composite membrane separators and solid-state electrolytes. In this paper, we will focus just on the first two types – microporous membrane separators and non-woven separators. The most common materials used for the preparation of microporous separators are polyethylene (PE), polypropylene (PP), polyvinylidene fluoride (PVDF), polyacrylonitrile (PAN) and polymethyl methacrylate (PMMA) or their combinations. The most commonly used materials in the case of non-woven separators are glass fibers, PVDF and PAN. This article describes unusual combinations of polymers polyethersulfone (PESU) and polyvinylpyrrolidone (PVP).^16,19,24,25^ For the preparation of microporous membrane separators, the dry and wet processes are primarily employed. Both processes are based on extrusion and application of stretching in one or two directions to increase porosity and tensile strength. A major part of the separator cost is influenced by the manufacturing method because of the low cost of polyolefin-based materials. The dry process began with the melt-extrusion of polyolefin films and their annealing at a temperature close to the polymer's melting point, which starts crystal formation. The next step of the dry process is a combination of low and high temperature uniaxial stretching and subsequently heat treatment, which fixes the pores formed during uniaxial stretching. Separators prepared by the dry process have a typical slit-like shape after this treatment. In the case of the wet process, the first step is to prepare a mixture of polymers and plasticizers. Subsequently, a thin film is extruded at an elevated temperature. As prepared thin film is afterwards calendared until its final thickness. Finally, after solidification, the separator is washed with solvents to remove residual solvent and create micro-sized pores. A microporous membrane is stretched in two axes, and the final shape of the pores is different compared to membranes prepared by the dry process. Pores are round-like, and their distribution is more uniform. The advantage of the wet process is that it can be used for many types of polymers, and the addition of plasticizers improves the workability of the polymer. On the other side wet process is more complicated and more expensive due to the higher number of process steps.^20^ Microporous membrane separators can be additionally improved by surface coating with some nanoparticles, for example, based on aluminium or silicon oxides. Kim *et al.* report positive influence of Al_2_O_3_ coating on thermal stability and short circuit prevention, and cycle life compared to standard PE separator.^26^ Further improvement was achieved by optimization of ceramic slurry composed of Al_2_O_3_ and PVP on PE separator by Cheng *et al.*^27^ Incorporation of the PVP binder promotes stronger interfacial adhesion of heat-resistant Al_2_O_3_ ceramic powders onto the PE substrate, resulting in a pronounced improvement in the thermal stability of the optimized separator. A slurry consisting of polyetherimide (PEI), Al_2_O_3_ and PVP was coated on the side of the PE separator and applied in Li–S batteries by Liu *et al.*^28^ The film breakage temperature and anti-puncture strength of the prepared PE/PEI separator were improved compared to the commercial PE separator. Wang *et al.* fabricated ZrO_2_@polyimide nano-microspheres-coated PE separators *via* a complexation-hydrolysis process.^29^ The prepared separator showed improved thermal stability and wettability, negligible shrinkage, and good long-term cycling stability. The separator material can be further modified using other components, such as carbon-based materials. Kim *et al.* report the use of graphene oxide in combination with silicon oxide and the positive influence of separator coating by GO–SiO_2_ composite on the cycle performance of LiCoO_2_-based Li-ion battery.^30^ This modification can help improve the performance of new battery systems such as Li–S batteries or manganese-based high voltage cathodes. Shi *et al.* report the use of covalent triazine-based frameworks as an interlayer on the surface of the separator to prevent polysulfide shuttle, and thanks to this, increase the capacity and capacity retention of Li–S cell.^31^ A similar effect was described by Bui *et al.*, who used a graphene microsphere-coated separator to prevent polysulfide shuttle.^32^ The optimized modification of material and porous structure of polymer separator can prevent magnesium dissolution and shuttle to Li anode in the manganese-based high voltage cathodes, as reported by Shin *et al.*^33^ The same procedure for microporous membrane separator enhancement can be used in the case of non-woven separators. Wu *et al.* used an electrospun poly(vinyl alcohol)-melamine nonwoven separator dip-coated and tested in the cell in combination with the NMC811 cathode.^34^ The prepared composite separators showed higher electrolyte uptake, good wettability, high ionic conductivity, lower activation energy, enhanced mechanical strength, improved thermal stability, and stable electrochemical performance. A hybrid ultra-high molecular weight PE (UHMWPE) separator with silicon dioxide (SiO_2_) nanocomposite membrane was prepared *via* a sequential biaxial stretching process by Babiker *et al.*^35^ Incorporation of SiO_2_ effectively enhanced the fundamental physicochemical and electrochemical properties of UHMWPE separators, including their thermal stability, wettability, electrolyte uptake, ionic conductivity and electrochemical performance. An important class of electrolyte systems closely related to separators is gel polymer electrolytes (GPEs), which combine the advantages of conventional liquid electrolytes with those of polymer-based separators. Polymer electrolytes offer several benefits, including improved tolerance to electrode volume changes during electrochemical cycling and high mechanical flexibility, contributing to enhanced safety and effective suppression of dendritic growth. Moreover, they enable operation at elevated temperatures and help mitigate parasitic reactions between electrodes.^36,37^ Nevertheless, these systems still suffer from several limitations, including low ionic conductivity at room temperature, sluggish ion diffusion and transport kinetics, and the formation of non-conformal interfaces with the electrodes.^38^ The conventional composition of gel polymer electrolytes (GPEs) consists of two main components: a polymer matrix and a dissolved salt. Among the various synthesis approaches, *in situ* polymerization is one of the most commonly employed methods. In this process, a mixture of monomer, liquid electrolyte, and polymerization initiator is introduced into the cell and subsequently cured under specific conditions, such as UV irradiation, thermal treatment, or electron-beam exposure.^37,39^ Falco *et al.* present the application of UV treatment for the preparation of cross-linked polymer electrolyte, which exhibits higher ionic conductivity and higher Li^+^ transference number at room temperature.^40^ The same as in the case of microporous membrane separators, GPEs can be modified for special applications such as Li–S battery systems. Tu *et al.* reported a M-MOF (M = Mn, Ni, Co) and carbon surface modification of poly(vinylidene fluoride-*co*-hexafluoropropylene) (PVDF-HFP) gel polymer electrolyte.^41^ The electrolyte with a dual modification strategy can increase ionic conductivity, improve migration number and kinetics of the chemical reaction, leading to good cycling results and rate performance.

## Experimental

### Electrode preparation

The high-voltage cathode material LiCr_0.1_Ni_0.4_Mn_1.5_O_4_ was synthesized by mixing the following precursors: Li_2_CO_3_ (lithium carbonate) and MnCO_3_ (manganese(ii) carbonate), NiO_2_ (nickel(ii) oxide) and Cr_2_O_3_ (chromium(iii) oxide). These materials were mixed in stoichiometric amounts of 0.02 mol l^−1^. Three-stage annealing process was chosen for their synthesis. The chosen precursors were milled together for 4 h in the first step to refine them and mix them. The resulting mixture was annealed at 600 °C for 10 h in the first annealing step. The second step was annealing at 900 °C for 15 h. The last (third) step was annealing at 700 °C for 15 h.^42^ A mixture consisting of the prepared material, PVDF (polyvinylidene fluoride) binder and Super P carbon was mixed by a magnetic stirrer in a vial with NMP (*n*-methyl-2-pyrrolidone) as a solvent in these weight ratios: cathode material 80%, Super P 10%, PVDF 10%. The resulting mixture was deposited on an Al foil by a 200 µm coating bar, dried and, after the solvent was evaporated, pressed by the pressure of 5000 N cm^−2^. A disc with a diameter of 18 mm was cut out from the coated Al foil. The disc was inserted into an electrochemical cell El-Cell^©^ ECC-STD, and assembled in an argon atmosphere inside an MBraun glove box. The loading of the active mass of the electrodes was 3.2 ± 0.3 mg cm^−2^. A lithium metal disc was used as a material for the anode, and the electrolyte was 1.5 mol l^−1^ LiPF_6_ in EC (ethylene carbonate) : DMC (dimethyl carbonate) in the ratio 1 : 2 w/w, and the electrolyte was soaked into the selected separators. Electrolyte loading per active mass was 20 µl mg^−1^ in the case of polymer separators and 30 µl mg^−1^ in the case of glass fiber separator. All chemicals for the electrode slurry and electrolyte preparation were purchased from Sigma-Aldrich.

### Investigated separators

Four types of separators were selected for this study (Table 1). The thickness of the separators was measured using a micrometre. The first type is a standard laboratory separator made of glass fiber (GF/C) with a thickness of 160 µm. The second analysed separator is a commercial separator, Celgard 2400. The separator is made in the form of a single layer of PP (polypropylene) foil with a thickness of 27 µm. The third investigated separator was the Nafigate A separator. It is a non-commercial separator made of nanofibers based on PVP and PESU with a thickness of 31 µm. The last tested separator was the Nafigate B separator, which is similar to Nafigate A, a non-commercial separator made of nanofiber based on PVP and PESU. The thickness of the Nafigate B separator was 26 µm. Samples of the nanofiber separators manufactured by the Nafigate company were prepared on the laboratory equipment NanospiderTM NS LAB 500.

**Table 1:** List of used separators

Name of separator	Producer	Material	Type of separator	Thickness [µm]
Glass fiber	Whatman plc	Glass fiber (GF/C)	Non-woven	160
Celgard 2400	Celgard, LLC	PP	Microporous	27
Nafigate A	Nafigate Corp.	PESU, PVP	Electrospun	31
Nafigate B	Nafigate Corp.	PESU, PVP	Electrospun	26

### Characterization methods of the separators and cells

Initially, the separators were characterized to evaluate their properties using a selected analytical method. Electrolyte uptake was measured for Celgard, glass fiber and Nafigate separators with conventional electrolyte (1 mol l^−1^ LiPF_6_ in EC : DMC in the ratio 1 : 1 w/w). The separator discs with a diameter of 20 mm were immersed in the electrolyte for 30 min at room temperature. Weights were measured before and after immersion in to electrolyte. The electrolyte uptake was determined using the following formula.Uptake (%) = [(*W* − *W*_0_)/*W*_0_] × 100where *W*_0_ and *W* are the weights of the separator disc before and after the immersion into the electrolyte.

Shrinkage of the separators was analyzed by heating to 100 °C, 110 °C, 130 °C and 150 °C. The duration of the elevated temperature was set for 1 h. Samples of the separators in the form of discs with a 17 mm diameter were monitored continuously, and changes in their size were recorded. Thermal stability of the separators was studied by thermogravimetry analysis (TGA) in argon atmosphere in the range of 35 to 900 °C with a scan rate of 5 °C min^−1^. The last parameter used for comparison of the separators was the conductivity of the separator in combination with a conventional electrolyte (1 mol l^−1^ LiPF_6_ in EC : DMC in the ratio 1 : 1 w/w) by electrochemical impedance spectroscopy (EIS). This measurement was done in the electrochemical cell El-Cell – ECC-STD assembled in an argon atmosphere inside the MBraun glove box. The measurement was done at room temperature, 40 °C and 60 °C. All measurements were performed three times, and the values were then averaged.

Cells prepared with the selected type of separator were characterized by galvanostatic cycling at different C-rates and at increased temperature. A Biologic VMP3 potentiostat was used for galvanostatic measurements. Galvanostatic cycling was carried out in a potential window from 3.0 to 5.1 V *vs.* Li^+^/Li. First, two charging and discharging cycles were carried out each time during which the used charging and discharging currents were set to 60 mAh g^−1^ (related to the active mass weight), *i.e.* 0.5C if the expected capacity is 120 mAh g^−1^. The real value of the sample capacity was deducted from these two cycles. Subsequently, the long-term cycling was started. In the first step, a cell with the high-voltage LiCr_0.1_Ni_0.4_Mn_1.5_O_4_ cathode and with the selected separator was cycled thirty times at 0.5C current. It was subsequently cycled five times at 1C current, then five times at 2C current and then five times at 5C current. After the gradual increase of current followed its gradual reduction. In the first step, there was cycling again five times at 2C current, then again five times at 1C current. After these cycles, the current load was set back to the original value of 0.5C and then twenty cycles at this load were performed. The tested cells were, after these twenty cycles, inserted into an oven heated to 55 °C where it was left for 30 min to stabilize at elevated temperature. Subsequently, cycling for twenty cycles at 0.5C current by the constant current-constant voltage (CCCV) method was started, where charging by the CV method was terminated when the current dropped below 1/10 of the original value. This method of cycling was mentioned in the Markovsky *et al.* article^43^ as suitable for cycling at high temperatures. Electrochemical impedance spectroscopy (EIS) of the cells was investigated during cycling by applying an AC voltage with an amplitude of 10 mV in the frequency range of 100 mHz to 1 MHz. EIS spectra were fitted using MATLAB software and the Zfit function.

All the investigated separators were analyzed using a scanning electron microscope (SEM) before and after cycling. An assembly consisting of the SEM microscope TESCAN VEGA3 XMU and a Bruker EDAX analyser was used to study separators and the created cathode material. Elemental mapping of the electrode material was investigated by energy-dispersive X-ray spectroscopy (EDS). X-ray photoelectron spectroscopy (XPS) was performed on a Kratos Ultra DLD spectrometer to confirm the composition of the created material.

## Results and discussion

One of the important parameters of the separator is electrolyte uptake, which has an influence on ionic conductivity and wetting time after cell assembly. Table 2 shows electrolyte uptake of the Celgard, glass fiber and uncommercial Nafigate separators, respectively. As summarized in Table 2, the electrospun Nafigate separators exhibit substantially higher electrolyte uptake than the commercial Celgard 2400 separator. In particular, Nafigate A and Nafigate B show electrolyte uptake values of 1655.2% and 1272.4%, respectively, compared to only 104.5% for Celgard 2400 and 717.6% for glass fiber. Electrolyte uptake of Celgard separator 104.5% is similar to electrolyte uptake presented by Uddin *et al.* 121%.^44^ This large difference originates from the highly porous, interconnected PESU/PVP nanofibrous structure, which provides abundant micro-/nanopores and strong capillary forces, enabling rapid electrolyte infiltration and efficient electrolyte retention.

**Table 2:** Electrolyte uptake results of the Celgard, glass fiber and uncommercial separators Nafigate

Name of separator	Weigh of separator [mg]	Weigh of separator after immersion [mg]	Electrolyte uptake [%]
Glass fiber	17.0	139.0	717.6
Celgard 2400	4.4	9.0	104.5
Nafigate A	2.9	50.9	1655.2
Nafigate B	2.9	39.8	1272.4

Although Nafigate A exhibits a higher electrolyte uptake than Nafigate B, the slightly lower uptake of Nafigate B suggests a more balanced fiber density and pore-size distribution, which helps maintain sufficient electrolyte retention while improving mechanical integrity and dimensional stability during cycling. This optimized microstructure facilitates continuous ion-transport pathways with reduced tortuosity, contributing to the superior cycling stability and high-rate performance observed for Nafigate B. However, excessively high electrolyte uptake may also reflect an overly open pore structure, which can adversely affect mechanical robustness and ion-transport uniformity during long-term cycling.

**Fig. 1:**
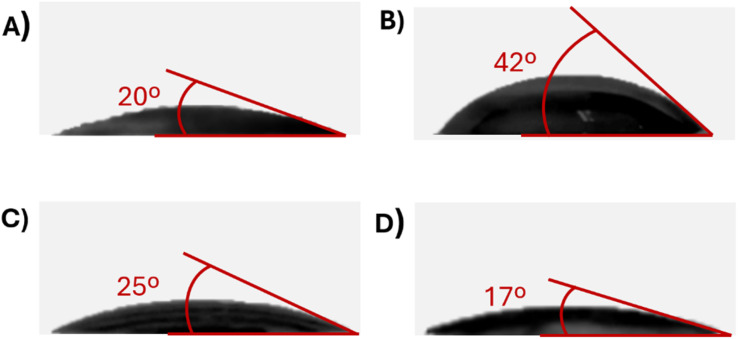
Pictures of the wettability of the separator: (A) glass fiber, (B) Celgard 2400, (C) Nafigate A, (D) Nafigate B immediately after the electrolyte drop.

The wettability test of all separators is shown in Fig. 1, and images were taken immediately (∼1 s) after drop casting of electrolyte on the surface of the separator. As can be seen from Fig. 1B Celgard 2400 showed the lowest wettability and the largest contact angle (42°). However, the highest wettability was shown by glass fiber (Fig. 1A) and Nafigate B (Fig. 1D) separators, the contact angles were 20° and 17°, respectively. The electrolyte was absorbed almost immediately for both separators. The contact angle of Nafigate A separator (25°) was slightly higher than that for Nafigate B.

**Fig. 2:**
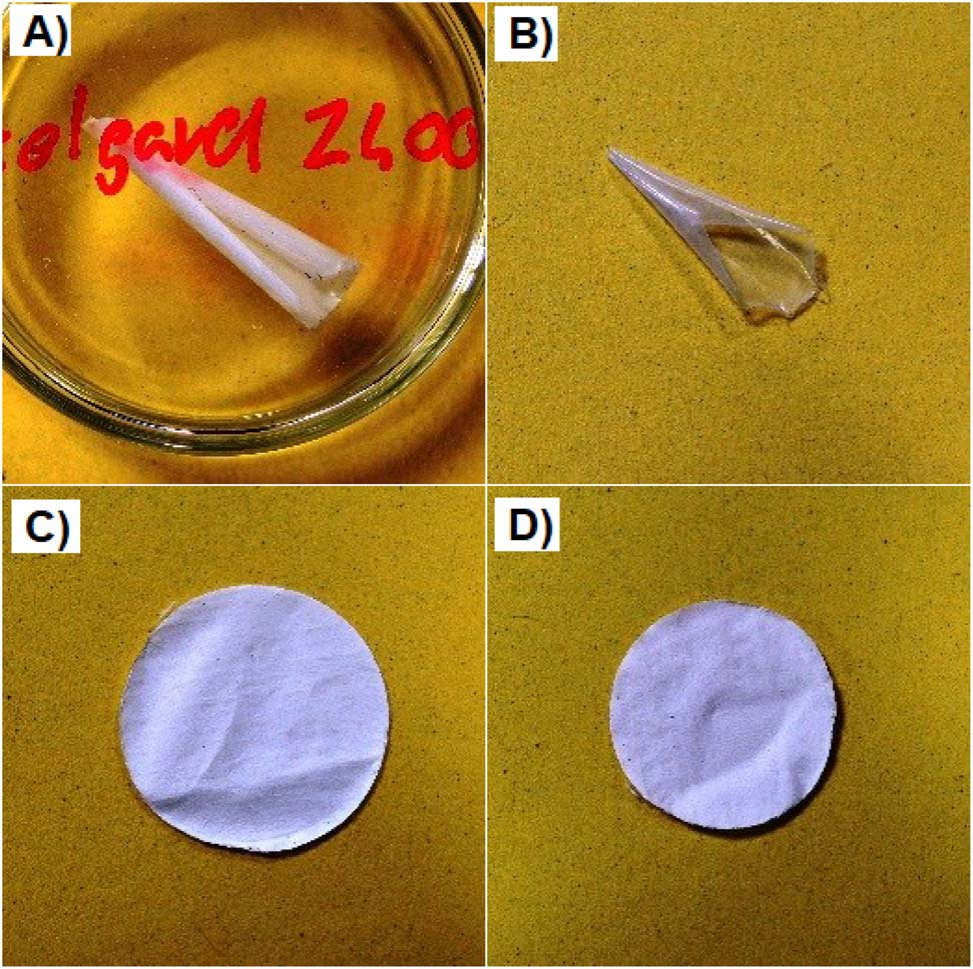
Pictures of the separators; (A) Celgard 2400 at room temperature, (B) Celgard 2400 after 2 h at 150 °C, (C) Nafigate B at room temperature, (D) Nafigate B after 2 h at 150 °C.

**Fig. 3:**
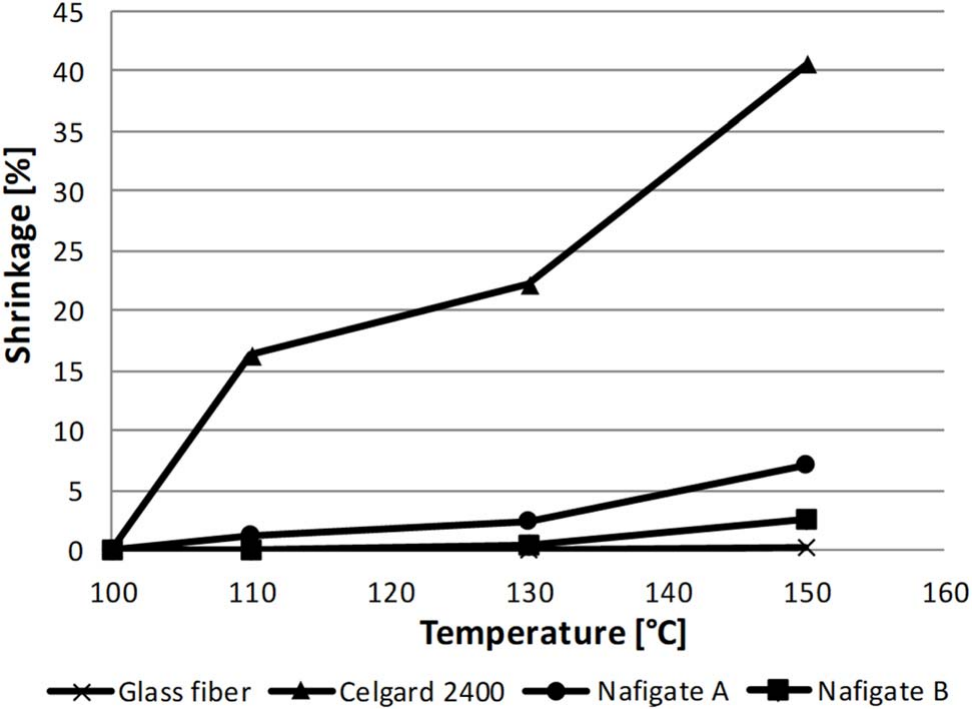
Dependence of the shrinkage on the temperature for all separators.

The separator Celgard 2400 before and after exposure to a temperature of 150 °C for 1 h is illustrated in Fig. 2A and B. Fig. 2C and D show the separator Nafigate B before and after exposure to the temperature of 150 °C for 1 h. The comparison of all separators shrinkage is shown in Fig. 3. According to expectations, the most dimensionally stable separator was the glass fiber separator because even at 150 °C, no shrinking was observed. The Nafigate separators were generally more stable than the commercial separator Celgard 2400. Nafigate B did not show any shrinkage at 110 °C, and the separator Nafigate A shrank just by 1.2%. The separator Celgard 2400 was much less dimensionally stable; it shrank by 16.3% at 110 °C and by 40.6% at 150 °C. The shrinking of the separator Nafigate A was 7.1% at 150 °C and it was only 2.5% for Nafigate B. The results observed in the case of Celgard 2400 separator are in correlation with the data reported by Yu *et al.*^45^ when they measured 16% shrinkage at 130 °C in the case of a PP membrane separator.

**Fig. 4:**
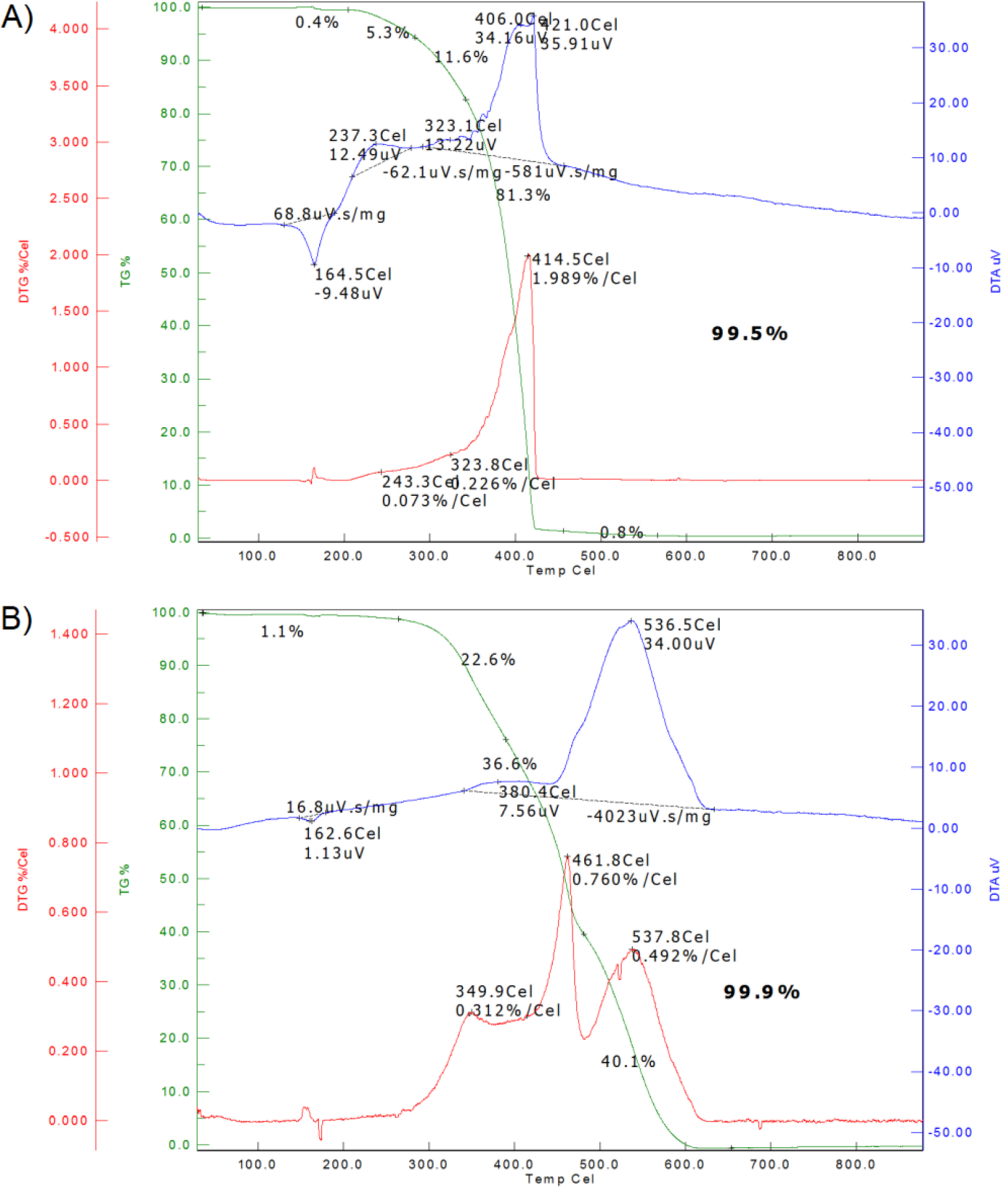
TGA analysis of the separators; (A) Celgard 2400 and (B) Nafigate B.

Another important parameter of separators is their thermal stability, which determines the highest temperature at which they can maintain their primary function of separating the positive and negative electrodes before disintegration. This property of the separator can be studied by the TGA (thermogravimetric analysis) analysis. The results from the TGA analysis of the Celgard 2400 separator are shown in Fig. 4A. On the TGA curve, it is visible that it is stable up to 170 °C, then begins a slow decrease in weight. The weight loss becomes very fast around 400 °C, and the overall weight loss past 500 °C is 99.5%. In the differential thermal analysis (DTA) curve, the first endothermic peak was observed at 164 °C, connected to the melting point of the separator closing the porous structure of the separator. The second exothermic peak at 406 °C is connected to the decomposition of the Celgard 2400 separator. The height of this peak is 35.91 µV. The TGA analysis of the separator Nafigate B is shown in Fig. 4B, where the weight of the separator is approximately constant up to 280 °C. This separator is therefore more thermally stable than the Celgard 2400 separator, which corresponds to the results observed during the shrinking measurement. The weight starts to decrease faster from 350 °C, and the separator is completely decomposed past 600 °C; overall weight loss was 99.9%. If we compare the data from the DTA analysis of the separator Nafigate B with the separator Celgard 2400, the first endothermic peak at 163 °C can be observed. However, this peak is lower than in the case of the Celgard 2400 separator. The second endothermic peak is split into two parts. The first is low, at 380 °C, and a higher one follows at a temperature of 537 °C. This temperature is higher, and the peak is lower (34 µV) compared to the separator Celgard 2400.

**Fig. 5:**
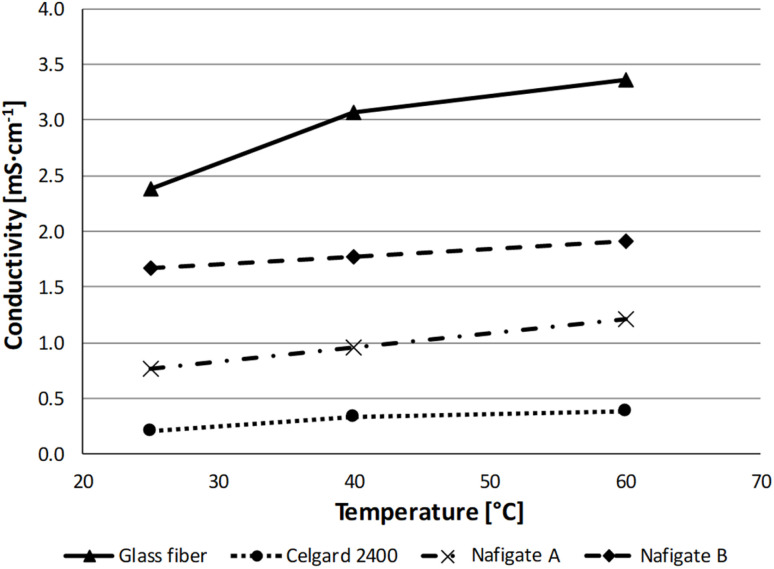
Conductivity of the separators at different temperatures.

The last analysis of the separators alone was the measurement of the conductivity of a separator impregnated with the electrolyte (1 mol l^−l^ LiPF_6_ in EC : DMC in the ratio 1 : 1 w/w) by EIS at room temperature, 40 °C and 60 °C. In Fig. 5, a comparison of the conductivity for all the separators is shown. The conductivity of the Celgard 2400 separator was in the range from 0.20 to 0.38 mS cm^−1^. The Nafigate separators exhibit higher conductivity, in the range from 0.77 to 1.21 mS cm^−1^ for Nafigate A and 1.67 to 1.91 mS cm^−1^ in the case of Nafigate B. The highest conductivity throughout the temperature range was measured for the glass fiber separator. Its conductivity was in the range from 2.38 to 3.37 mS cm^−1^.

Although the Nafigate separators exhibit a markedly higher electrolyte uptake (Table 2), their ionic conductivity (Fig. 5) remains lower than that of the glass fiber separator, indicating that electrolyte uptake alone does not govern ion transport; instead, effective conductivity is strongly influenced by pore connectivity, transport tortuosity, and partial immobilization of electrolyte within polymer-rich domains.^46,47^

### Characterization of the separators with cathode materials and characterization of the cathode material

**Fig. 6:**
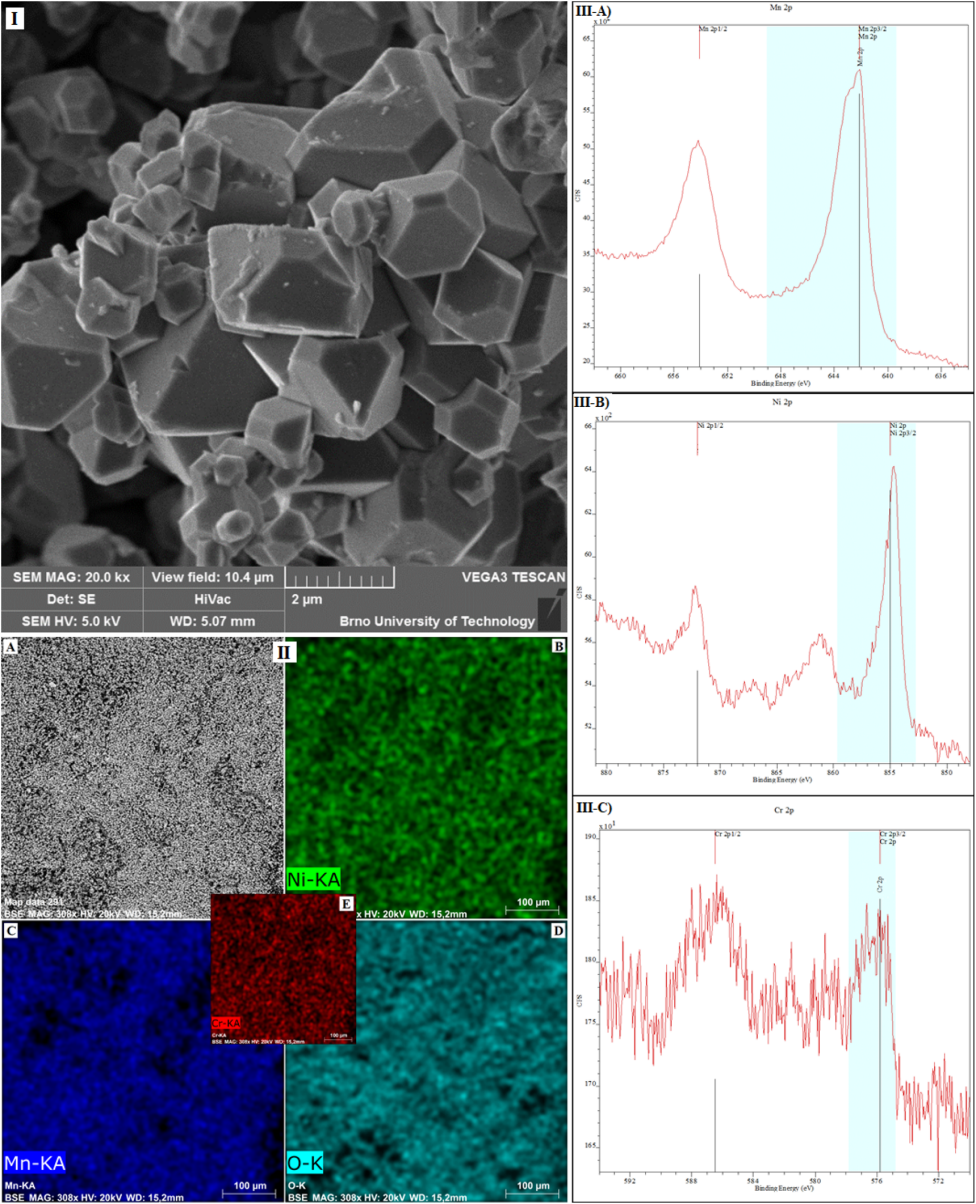
(I) SEM analysis of the LiCr_0.1_Ni_0.4_Mn_1.5_O_4_ material after synthesis, used field of view 10.4 µm; (II) mapping of the sample LiCr_0.1_Ni_0.4_Mn_1.5_O_4_; (A) SEM particles of LiCr_0.1_Ni_0.4_Mn_1.5_O_4_, (B) distribution of nickel, (C) distribution of manganese, (D) distribution of oxygen, (E) distribution of chromium; (III) XPS spectroscopy of the LiCr_0.1_Ni_0.4_Mn_1.5_O_4_ sample, (A) Mn 2p, (B) Ni 2p, (C) Cr 2p.

To investigate the morphology of the synthesized cathode material LiCr_0.1_Ni_0.4_Mn_1.5_O_4_, SEM analysis was performed and is shown in Fig. 6-I (field of view 10.4 µm). The aggregates of small crystals in the entire volume are present, and the size of the crystals is less than 3 µm. The EDS analysis with surface mapping of the sample was performed together with the SEM analysis. The EDS mapping of the distribution of the elements in the LiCr_0.1_Ni_0.4_Mn_1.5_O_4_ sample can be seen in Fig. 6-II. Fig. 6-II, which confirms the demanded uniformity of the distribution of Mn, Ni, Cr and O in the synthesized LiCr_0.1_Ni_0.4_Mn_1.5_O_4_ active material.

The elemental composition of the synthesized cathode material LiCr_0.1_Ni_0.4_Mn_1.5_O_4_ was investigated by XPS analysis, and the resulting spectra are shown in Fig. 6-III. In Fig. 6-III-A is the spectrum of Mn 2p, and the spectrum of Ni 2p is in Fig. 6-III-B. Ni peaks are in the places with the bond energy of 854.5 eV for Ni 2p_1/2_ and 872.1 eV for Ni 2p_3/2_. They (together with the satellite peaks) represent Ni in the Ni^2+^ valence state. The main Mn peak – Mn 2p_3/2_ is at 642.1 eV, and the Mn 2p_1/2_ peak is at 654.0 eV. The main peak (Mn 2p_3/2_) confirms the presence of Mn in the Mn^4+^ oxidation state, which corresponds to findings of previous studies.^48^ The spectrum indicates the presence of chromium in Fig. 6-III-C, which, according to the peak positions at 576 eV, suggests the presence of the element chromium in the Cr^3+^ state.^49^

**Fig. 7:**
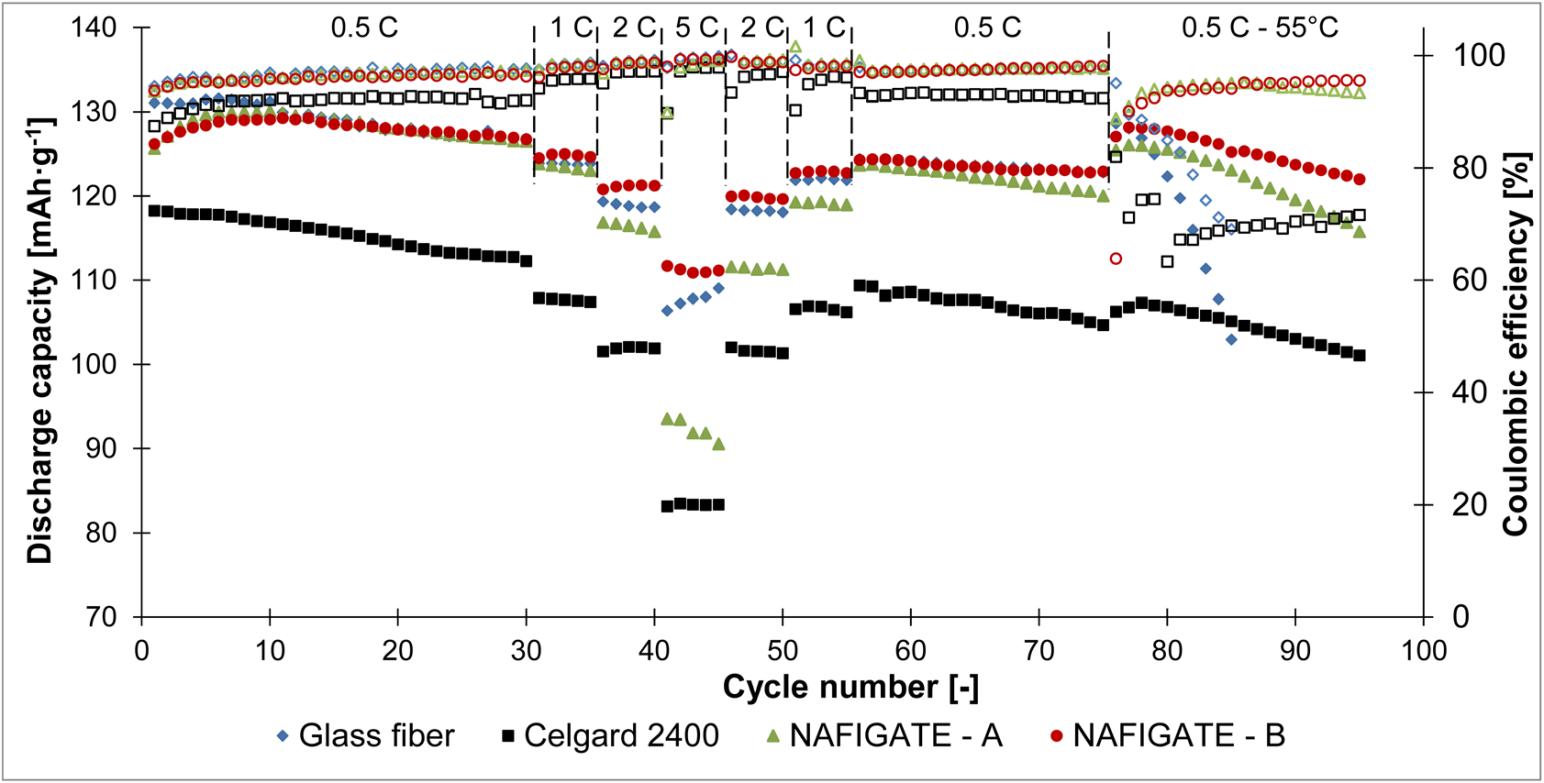
Comparison of the capacity and coulombic efficiency during cycling at different C-rates and different temperatures of the LiCr_0.1_Ni_0.4_Mn_1.5_O_4_ sample in combination with the glass fiber, Celgard 2400, Nafigate A and Nafigate B separators.

Fig. 7 shows the cycling of all tested separators in combination with the high voltage cathode material LiCr_0.1_Ni_0.4_Mn_1.5_O_4_ recorded over 90 cycles at different C-rates and at different temperatures.

A gradual increase in capacity occurred during the first cycles at 0.5C for the cells with Nafigate A and Nafigate B separators. Capacities achieved by the cells with the glass fiber separator, Nafigate A and Nafigate B separators after the tenth cycle were comparable. The cell with the glass fiber separator reached the highest capacity (131.1 mAh g^−1^) at the beginning of cycling. In contrast, the lowest capacity and the largest capacity decrease during the first thirty cycles at 0.5C showed the cell with the commercial separator Celgard 2400. Its capacity during these thirty cycles decreased by 5.1%. The cell with the Nafigate B separator showed a slight increase in capacity (0.4%) compared to the first cycle at 0.5C. The increase in capacity of the cell with the Nafigate A separator was very similar (about 0.7%). The cell with the glass fiber separator showed a decline in capacity of about 3.3% during the first thirty cycles at 0.5C. A decrease in capacity was observed for all the tested cells after the load was increased to 1C. Cells using the glass fiber separator and the Nafigate B separator were highly stable during these five cycles by 1C current. However, the cells using separators Celgard 2400 and Nafigate A showed only a negligible capacity decrease (about 0.4% and 0.6% for the Nafigate A separator). The achieved capacities decreased again after another load increase to 2C. The most stable cell at this load was again the one with the Nafigate B separator. No capacity decrease was observed after these five cycles. This cell is also the most stable in terms of capacity decrease in response to increasing load. The capacity value in the last cycle at 2C current was equal to 98.8% of the first cycle capacity of the overall cycling. The least stable cell at this load was the one with the Celgard 2400 separator, whose capacity at the end of the cycling at 2C was equal to 90.2% of the first cycle capacity. There are visible large differences between the cells at the highest load (5C). The highest capacity was reached by the cell with the Nafigate B separator, and the lowest again for the cell with the Celgard 2400 separator, which, in the last cycle at the maximum load, reached 70.0% of the first cycle capacity of the entire cycling. The cell with the Nafigate B separator reached 88.0% of its initial value. The capacity of the cell with the glass fiber separator decreased to 83.2% of the initial capacity, and it was 72.1% in the case of the cell with the Nafigate A separator. The load was reduced to the initial value of 0.5C during the subsequent cycles. All tested cells returned approximately to the same capacity values as at the beginning of the experiment. The only exception was the cell with the Nafigate A separator, which showed significantly lower capacity than during the previous cycling under repeated load at 2C and 1C. The most stable cell during repeated cycling at 0.5C was the cell with the Nafigate B separator, after seventy-five cycles at various loads, showing a capacity decline of about 2.6% compared to the first cycle. The cell with Nafigate A showed a capacity decrease of 4.5% and it was 6.3% in the case of the cell with the glass fiber separator. The highest capacity decline was the cell with the Celgard 2400 separator (about 11.5%).

In addition, rate capability test at room temperature was followed by twenty cycles at 0.5C at a temperature of 55 °C. All the tested cells showed an increase in capacity at the beginning of cycling caused by the increased temperature facilitating electrochemical reactions and by the CCCV charging regime. After this initial increase in capacity, the cell with the glass fiber separator showed a sharp decline in capacity during a few cycles, and the cell was then disconnected. Nevertheless, the rapid capacity decay and unstable cycling behavior observed for the glass fiber separator at 55 °C can be explained based on well-established electrolyte decomposition and separator interaction mechanisms reported in the literature.^41^ At elevated temperatures, carbonate-based electrolytes undergo accelerated thermal decomposition, producing reactive species such as HF, POF_3_, and other acidic by-products derived from salt (*e.g.*, PF_6_^−^) degradation. These species are known to strongly interact with inorganic, silica-based glass fiber separators, leading to surface corrosion, formation of insulating residues, and increased interfacial resistance. HF generated at high temperatures can react with silicate components in glass fiber materials, forming fluorosilicates and releasing water, which further promotes electrolyte decomposition and parasitic reactions. Such chemically driven degradation processes are accompanied by accumulation of decomposition residues on the separator surface, pore blockage, and loss of effective ion transport, ultimately resulting in rapid capacity fading and premature cell failure under thermal stress. In contrast, the polymer-based Nafigate separators exhibit significantly improved stability at 55 °C, indicating that the failure of the glass fiber separator is closely related to its unfavorable chemical interaction with electrolyte decomposition products rather than solely mechanical factors.

The most stable cell was the one with the Nafigate B separator. The decrease in capacity for this cell was 5% after the whole cycling, *i.e.* after ninety-five cycles. The cell using the Nafigate A separator lost 7.9% of its capacity. The least stable cell (not considering the cell with the glass fiber separator) was the one with the Celgard 2400 separator capacity decreased by 17.7%. A comparison of the Coulombic efficiencies achieved by individual cells shows that the lowest efficiency at room temperature was achieved by cell with Celgard 2400 separator. The efficiencies of the Nafigate A, B and Glass fiber separators were very similar during cycling at room temperature. Significant differences were then observed during cycling at high temperatures, when all cells experienced a decrease in efficiency. In the case of the cell with a glass fiber separator, there was a gradual loss of efficiency with each cycle, with efficiency falling from 95.1% to 69.0% after ten cycles. In the case of the cell with the Celgard 2400 separator, the efficiency gradually decreased to 63.3% and then slowly increased to 71.6%. The cells with the Nafigate A and B separators showed a decrease in the first cycle at 55 °C and then an increase. For the cell with the Nafigate B separator, this decrease in the first cycle at 55 °C was more pronounced, but the efficiency subsequently increased to 95.6%. Which was higher than efficiency for the cell with the separator Nafigate A. The improvement in the stability of the high-voltage cathode is also evident not only in the higher capacities achieved and the smaller decrease in capacity, but also in the higher efficiency achieved.

**Fig. 8:**
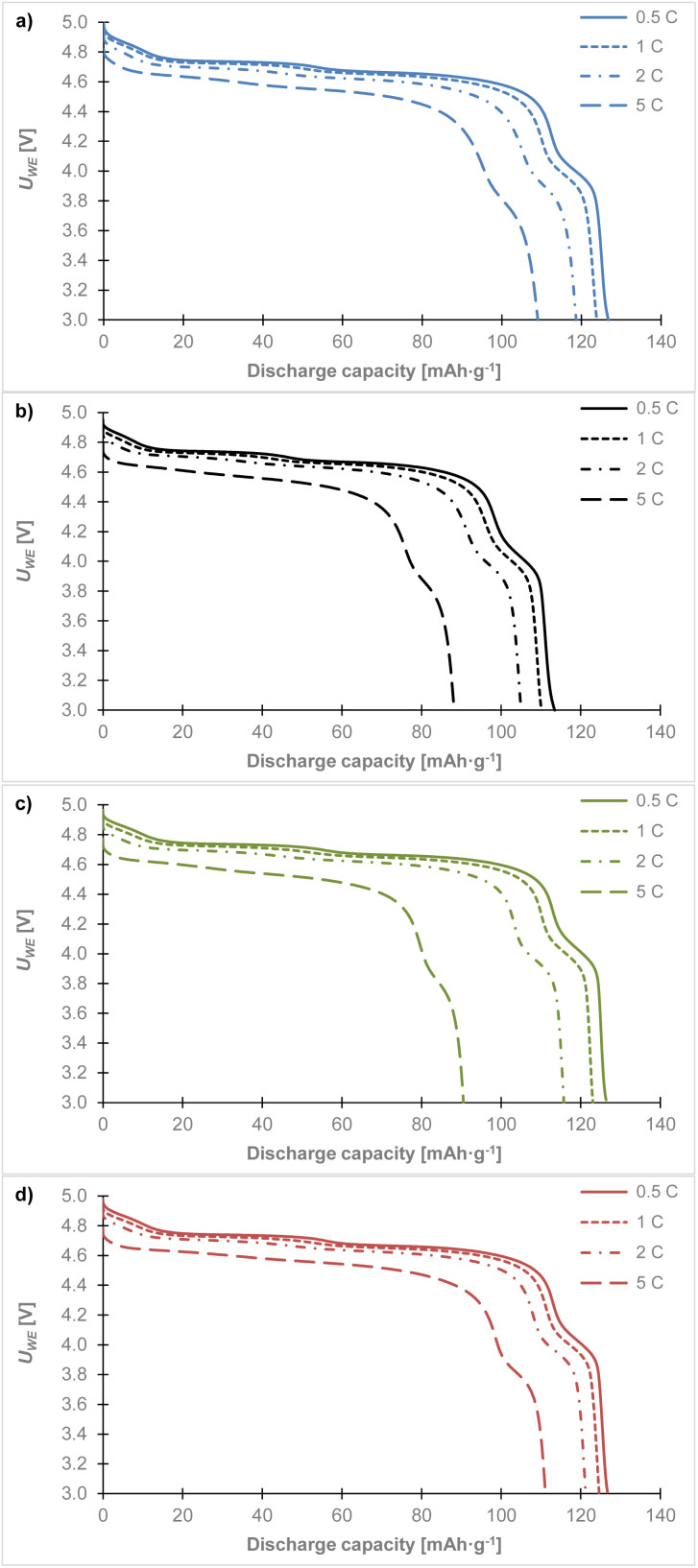
Comparison of discharge curves for the LiCr_0.1_Ni_0.4_Mn_1.5_O_4_ sample at different C-rates in combination with separator: (a) glass fiber, (b) Celgard 2400, (c) Nafigate A, (d) Nafigate B. Data was taken from the last cycle at the corresponding C-rate.

Fig. 8 shows the discharge curves from the last cycles at different C-rates. The high voltage cathode material LiCr_0.1_Ni_0.4_Mn_1.5_O_4_ in combination with (a) the glass fiber, (b) the Celgard 2400, (c) the Nafigate A, and (d) the Nafigate B separator. As can be seen, each combination of the cathode and separator showed a stable high voltage plateau which began close to 4.9 V. This high voltage plateau is quite stable for all the cells at approximately the same potential until a 2C rate. There is a clear drop in voltage of this plateau close to 4.6 V at 5C. Comparing the capacity achieved at the high potential plateau (up to 4.4 V) with the discharged capacity at 5C, shows that the cell with the Nafigate B separators reached 79.4% of the capacity at the high potential of up to 4.4 V. The corresponding values were 77.7% in the case of the Nafigate A separator and 77.4% for the glass fiber separator. The lowest high voltage plateau capacity was achieved by the Celgard 2400 separator; it was 76.8% at 5C. The results show that the cell with the Nafigate B separator was able to deliver the highest power at a higher load compared to the other investigated separators. The second most stable cell at 2C load was the cell with the Nafigate A separators, where the capacity at the end of cycling at 2C was equal to 97.9% of the capacity in the first cycle of the overall cycling. Overall, the Nafigate B separators deliver stable mos stable cycle performance and the curves are without short-circuit signatures.

Considering the thickness of the separators, the glass fiber separator is substantially thicker (160 µm) than the others, which likely increases ionic resistance and contributes to its lower rate performance and cycling stability. Conversely, the Nafigate B separator combines one of the thinnest profiles (26 µm) with a highly porous, nanofibrous architecture, facilitating shorter ion transport pathways and improved electrolyte wettability. While thickness is an important factor, it does not act in isolation. Porosity, pore size distribution, electrolyte uptake, and surface chemistry also strongly influence ionic conductivity and interfacial stability. The electrospun PESU/PVP separators (Nafigate A and B) demonstrate that a well-optimized microstructure can compensate for slight differences in thickness, yielding superior overall performance compared to commercial separators with thicker or microporous morphologies.

**Fig. 9:**
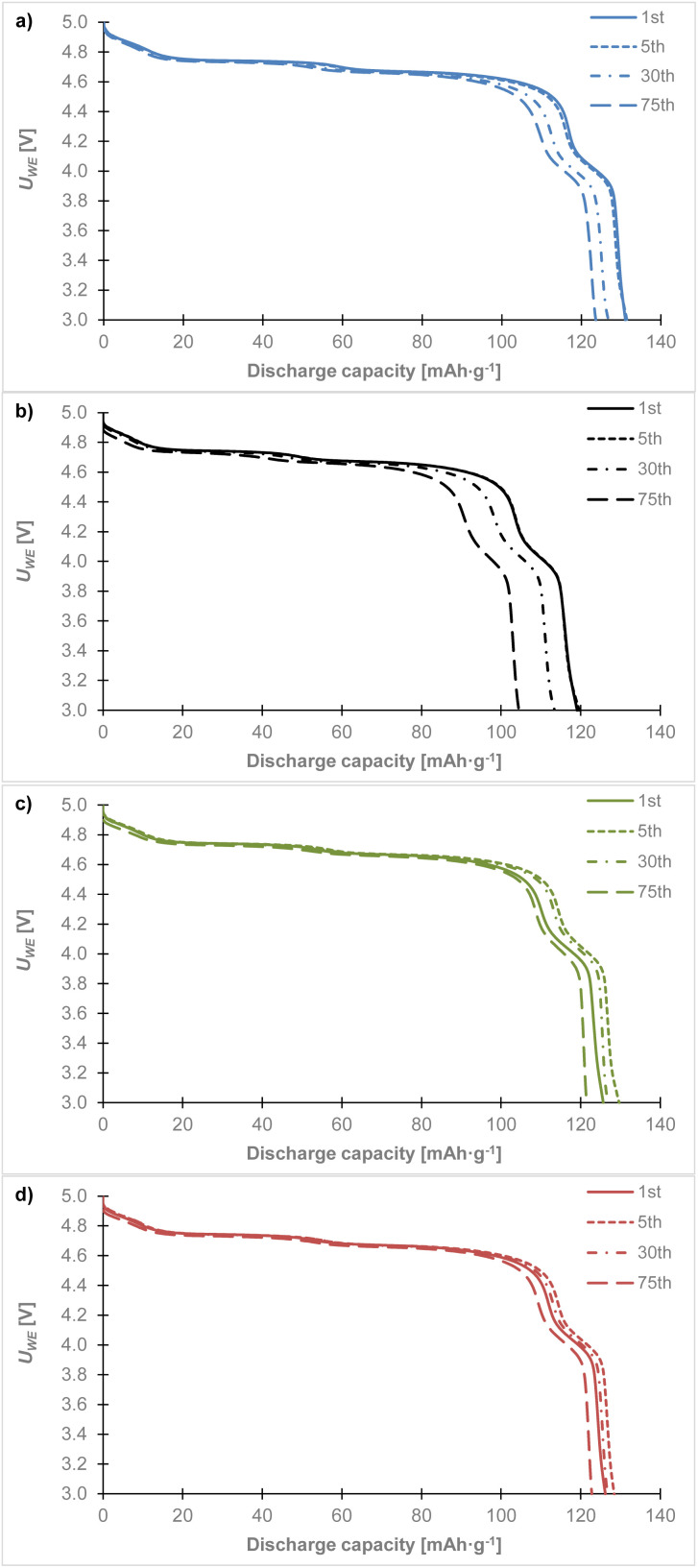
Comparison of discharge curves for the LiCr_0.1_Ni_0.4_Mn_1.5_O_4_ sample at 1st, 5th, 30th and 75th cycle in combination with separator: (a) glass fiber, (b) Celgard 2400, (c) Nafigate A, (d) Nafigate B.

Fig. 9 shows discharge curves for the 1st, 5th, 30th, and 75th cycles. Cell with Celgard 2400 separator (Fig. 9b) is most unstable. During cycling, a gradual decrease in capacity is noticeable, with a significant decrease between the 5th and 30th cycles and a similar decrease between the 30th and 75th cycles. At the same time a voltage drop in the upper discharge plateau is evident in the last 75th cycle. Cell with a glass fiber separator (Fig. 9a) had almost identical discharge curves in the 1st and 5th cycles. The capacity in the first cycle of 131.1 mAh g^−1^ corresponded to 88.6% of the theoretical capacity. However, between the 5th and 30th cycles, we observed a capacity drop and a similar capacity drop can be seen between the 30th and 75th cycles. In the 75th cycle, a capacity of 122.8 mAh g^−1^ was achieved, which corresponds to 83% of the theoretical capacity. This represents capacity decrease of 5.6% compared to the theoretical capacity. The cell with the Nafigate A (Fig. 9c) separator showed an increase in capacity compared to the first cycle. Between the 5th and 30th cycles, there was a slight decrease, however, the capacity remained higher than in the first cycle. Between the 30th and 75th cycles, capacity decreased more significantly than in the cell with a glass fiber separator. The potential of the upper discharge plateau changed only slightly. The cell with the Nafigate B separator showed the highest stability. In the first cycle, a capacity of 126.2 mAh g^−1^ was achieved, which corresponds to 85.3% of the theoretical capacity. The capacity at the 5th and 30th cycles was higher than at the first cycle, and at the same time, the decrease between the 30th and 75th cycles was less significant than in the cell with the Nafigate A separator. In the 75th cycle, a capacity of 122.9 mAh g^−1^ was achieved, which corresponds to a decrease of 2.2% compared to the theoretical capacity.

**Fig. 10:**
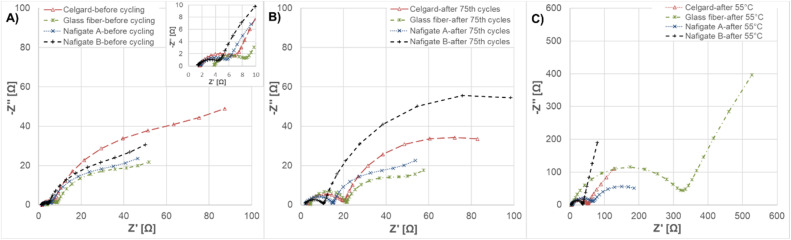
Electrochemical impedance spectroscopy of Li/LiCr_0.1_Ni_0.4_Mn_1.5_O_4_ cell with Celgard, glass fiber, Nafigate A and Nafigate B separators: (A) before cycling, (B) after 65th cycles, (C) after cycling at 55 °C.

**Fig. 11:**
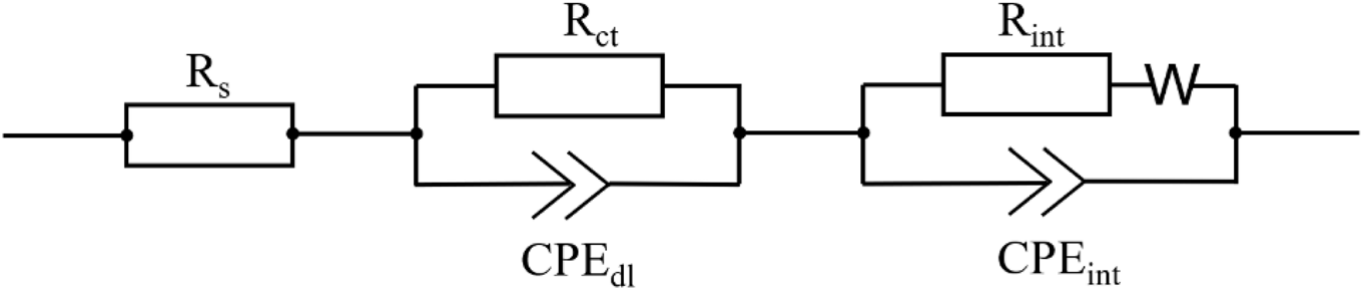
Equivalent electrochemical circuit used for fitting of EIS spectra for all separators.

Nyquist plots of the Li/LiCr_0.1_Ni_0.4_Mn_1.5_O_4_ cells with glass fiber, Celgard 2400, Nafigate A and Nafigate B separator at different states of cycling are shown in Fig. 10. Applied equivalent electrochemical circuit for EIS fitting is illustrated in Fig. 11. Fig. 10A shows electrochemical impedance spectroscopy (EIS) before cycling. Charge transfer resistance (*R*_ct_) of the cell with Nafigate B separator is the lowest (∼3.5 Ω). The increasing *R*_ct_ was observed for Nafigate A (∼4.4 Ω) and glass fiber separator (∼5.0 Ω). The highest *R*_ct_ before cycling was observed in the case of the cell with Celgard 2400 separator (∼5.7 Ω). Fig. 10B shows EIS after the 75th cycle. All cells show an increase in *R*_ct_; however, the lowest value of ∼9.6 Ω and percentage increase was observed in the cell with Nafigate B separator. After cycling at 55 °C (Fig. 10C) was a considerable change in *R*_ct_, mainly for the cell with glass fiber separator. The *R*_ct_ value increased to ∼321 Ω, which is more than sixty times higher value. The lowest *R*_ct_ after cycling at 55 °C was observed for the cell with Nafigate B separator (∼33.3 Ω). All these results are in agreement with results from previous analysis. The cell with Nafigate B separator has better electrochemical properties and lower resistance thanks to high structural stability, high wettability and high conductivity, respectively. The cell with this separator can work more stably at high C-rate and at high temperature (Table 3).

**Table 3:** *R*_s_ and *R*_ct_ values before cycling, after 75th cycle, after cycling at 55 °C for all investigated separators

Name of separator	Before cycling		After 75th cycle		After cycling at 55 °C	
	*R*_s_ [Ω]	*R*_ct_ [Ω]	*R*_s_ [Ω]	*R*_ct_ [Ω]	*R*_s_ [Ω]	*R*_ct_ [Ω]
Glass fiber	3.7	5.0	4.3	17.2	5.8	321.2
Celgard 2400	1.6	5.7	2.3	18.5	3.5	48.2
Nafigate A	1.7	4.4	1.8	13.5	4.6	63.1
Nafigate B	1.3	3.5	1.3	9.6	3.7	33.3

**Fig. 12:**
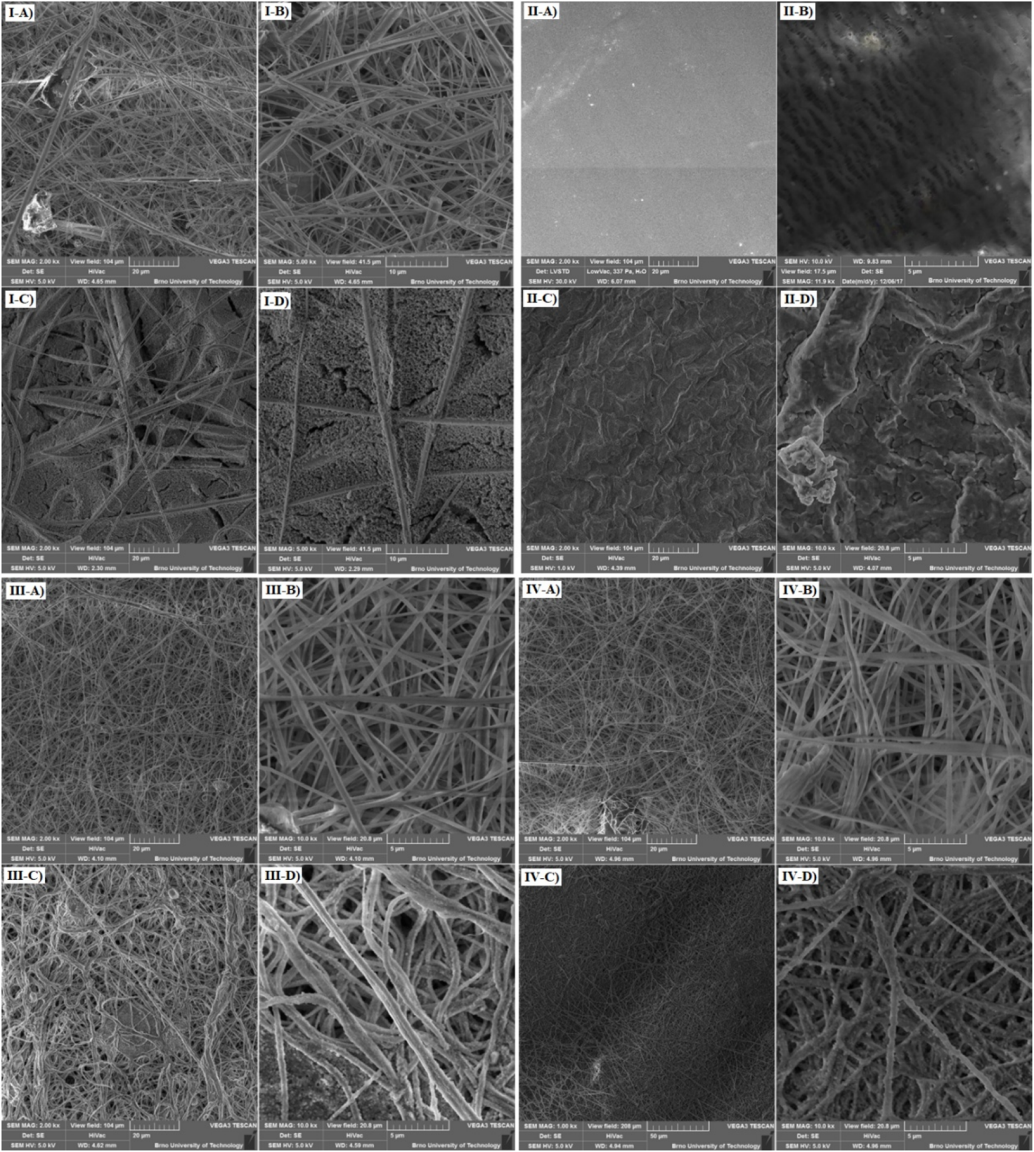
(I) SEM analysis of the glass fiber separator; (A) before cycling, used field of view 104 µm, (B) before cycling, used field of view 41.5 µm, (C) after cycling, used field of view 104 µm, (D) after cycling, used field of view 41.5 µm, (II) SEM analysis of the Celgard 2400 separator; (A) before cycling, used field of view 104 µm, (B) before cycling, used field of view 17.5 µm, (C) after cycling, used field of view 104 µm, (D) after cycling, used field of view 20.8 µm; (III) SEM analysis of the Nafigate A separator; (A) before cycling, used field of view 104 µm, (B) before cycling, used field of view 20.8 µm, (C) after cycling, used field of view 104 µm, (D) after cycling, used field of view 20.8 µm; (IV) SEM analysis of the Nafigate B separator; (A) before cycling, used field of view 104 µm, (B) before cycling, used field of view 20.8 µm, (C) after cycling, used field of view 208 µm, (D) after cycling, used field of view 20.8 µm.

The used separators were studied using SEM after cycling, and their morphology was compared with that of the pristine samples. All the observations were performed using secondary electrons at an acceleration voltage of 5 kV in vacuum. The glass fiber separator before and after cycling are shown in Fig. 12-I. There is a clearly visible fibrous structure of the separator before cycling in Fig. 12-I-A and I-B. It can be seen in the images after cycling (Fig. 12-I-C and I-D) that possible residues after the decomposition of the electrolyte settled in the spaces between the fibers of the separator. This could block the separator and affect the function of the battery. This phenomenon might have occurred during cycling at high temperature, which would explain the rapid decline in the capacity observed during this cycling and the significant increase in charge transfer resistance by EIS measurements.

The images of the Celgard 2400 separator before and after cycling are shown in Fig. 12-II. The smooth and uniform surface of the PP film before cycling is shown in Fig. 12-II-A. The structure of individual pores in the separator is shown in Fig. 12-II-B. The height of individual pores is about 0.5 µm, and their width is several µm. A clearly noticeable difference in the structure of the separator after cycling is visible in Fig. 12-II-C, which uses the same field of view as the previous figure. The separator after cycling is wrinkled, and upon closer inspection (see Fig. 12-II-D), many depressions and holes are visible on its surface. The separator was probably damaged during cycling at an elevated temperature in combination with cycling in a system with a higher potential.

The Nafigate A separator before and after cycling is shown in Fig. 12-III. This separator is a nanofiber separator with fiber thickness in hundreds of nanometers, see Fig. 12-III-A and III-B. The voids between the fibers of the separator after cycling (Fig. 12-III-C and III-D) were partially clogged in some areas; however, the fibers themselves, including their shape and size, remained largely preserved after cycling. In comparison, previous separators were only covered with a layer of sediment at certain spots.

The last analyzed separator was the Nafigate B separator whose nanofibrous structure is shown in Fig. 12-IV. The separator is, similarly to the Nafigate A separator, made of nanofibers, and the size of the fibers is in the range of hundreds of nanometers, see Fig. 12-IV-A and IV-B. However, the fiber density for this separator is lower than for the Nafigate A separator. After cycling (see Fig. 12-IV-C and IV-D), these fibers show that the structure was maintained after cycling, and there are no visible large clumps due to the decomposition of the electrolyte, as in the previous samples. The surface of the fibers is covered only with what seems to be its residues. Thanks to this, the separator was capable of ensuring sufficient passage of Li^+^ ions from one electrode to the other during cycling at different loads and higher temperature. There is no visible separator demage after cycling, suggesting that dendrite growth is not present under the studied conditions.

## Conclusions

The properties of two non-commercial separators, prepared by Nafigate, have been tested and then compared with two types of commercial separators (glass fiber and Celgard 2400). Both Nafigate separators exhibit a significant improvement regarding shrinkage. The thermal stability of the Nafigate B separator was also better than that of Celgard 2400. Improved thermal stability contributes to battery safety by shifting the exothermic decomposition of the separator to higher temperatures, thereby reducing the risk of thermal runaway. Moreover, the peak related to the exothermic reaction of the Nafigate separator was slightly lower. The Nafigate B separator also showed much higher conductivity compared to the Celgard 2400 separator, and its conductivity is close to the conductivity of the glass fiber separator. Electrolyte uptake and wettability are both significantly enhanced in Nafigate separators than in commercial Celgard and glass fiber separators. The second part of this article is focused on the influence of separators on the function of the battery with high-voltage cathode material LiCr_0.1_Ni_0.4_Mn_1.5_O_4_. Long-term galvanostatic cycling at different C-rates and cycling at elevated temperature were used to demonstrate that the used separator can greatly affect the function of a high-voltage battery. The least stable during all the cycling was the cell with the commercial separator Celgard 2400. This cell also reached the lowest capacity throughout the cycling. Lower capacities, especially at higher C-rates, are probably caused by the lower conductivity of the Celgard 2400 separator in combination with the electrolyte. It was also observed by SEM analysis that the originally perfectly flat PP foil was substantially damaged during cycling, and its structure was completely changed. Many holes and depressions were created on its surface, indicating its disintegration. Therefore, the results suggest that this commercially used separator seems to be rather inappropriate for the high voltage Li-ion systems.

Another interesting result was discovered by testing the conventional glass fiber separator, which showed almost the best results in terms of stability and achieved capacity practically throughout the whole cycling procedure. The only exception was cycling at a higher temperature, when a rapid loss of capacity occurred for the cell with this separator. This decrease can be explained by using the data obtained from SEM, where very large deposits, likely residues from the decomposition of electrolyte, can be seen between the glass fibers after cycling. This sedimentation most likely took place at the increased temperature when the electrolyte is more loaded and the cathode material is also more stressed because of the tendency of Mn to dissolve into the electrolyte at higher temperatures. There is most likely a reaction between the electrolyte, the cathode material and the surface of the separator at high temperature. Consequently, the separator is gradually blocked by the residues, which cause the loss of capacity during cycling.

The best results from the tested Nafigate company separators showed the Nafigate B separator. This separator, in combination with the LiCr_0.1_Ni_0.4_Mn_1.5_O_4_ cathode material, showed the highest long-term cycling stability at higher temperature and the highest capacity at high C-rates. This separator, based on PESU, PVP nanowires, provides very good thermal stability, low shrinkage, comparable conductivity with the glass fiber separator and high stability in combination with cathode materials with a high potential window. Wetting of the separator is comparable with the glass fiber separator; however, its electrolyte uptake is significantly higher. This is supported by the SEM analysis, where there were no major changes in the fibrous structure of this separator, and there was no deposition of residues from the decomposition of electrolyte in the space between the fibers. High-voltage Li-ion battery systems like the LiCr_0.1_Ni_0.4_Mn_1.5_O_4_ cathode materials are limited by electrolyte oxidation, transition-metal dissolution, interfacial instability, and increased thermal stress. The results of this study show that the Nafigate B separator effectively mitigates these challenges through its high thermal stability and low shrinkage, which enhance safety under high-voltage operation, and its high electrolyte uptake and wettability, which promote uniform Li^+^ transport and reduce localized overpotentials that accelerate electrolyte oxidation. The absence of significant structural degradation or electrolyte decomposition residues observed by SEM after cycling further indicates suppressed parasitic reactions, enabling stable cycling performance at 5.1 V and at elevated temperature.

## Conflicts of interest

There are no conflicts to declare.

## Data Availability

Data for this article, including data from electrochemical analysis are available on Zenodo at https://doi.org/10.5281/zenodo.17751117.
